# Supplementary material for: A multiple ion-uptake phenotyping platform reveals shared mechanisms affecting nutrient uptake by roots
Source: Plant Physiol. 2020 Dec 29;185(3):781–95. doi: 10.1093/plphys/kiaa080 (PMC8133564; doi:10.1093/plphys/kiaa080)
Supplement: kiaa080_Supplementary_Data [file kiaa080_supplementary_data.zip › pp.01265.2020-s02.docx]

**Supplemental Data**

**Supplemental Figure S1.** Genetic diversity among nested association mapping (NAM) population founder lines and broad group classification for specific phosphate uptake rates. Specific phosphate uptake rates from solution concentrations of (A) 25 μM and (B) 250 μM. (C) The net specific phosphate uptake rate ratio between the solutions, with a ratio >1 (dotted line) representing a greater uptake rate in the high concentration compared to the low concentration. The specific phosphate uptake rates by broad group classification in the solution concentrations of (D) 25 μM and (E) 250 μM. Boxplots show the median values (center horizontal lines), upper and lower quartiles (box length), minimum and maximum values (whiskers), mean values, (dots within whiskers), and, outliers (dots outside of the whiskers).


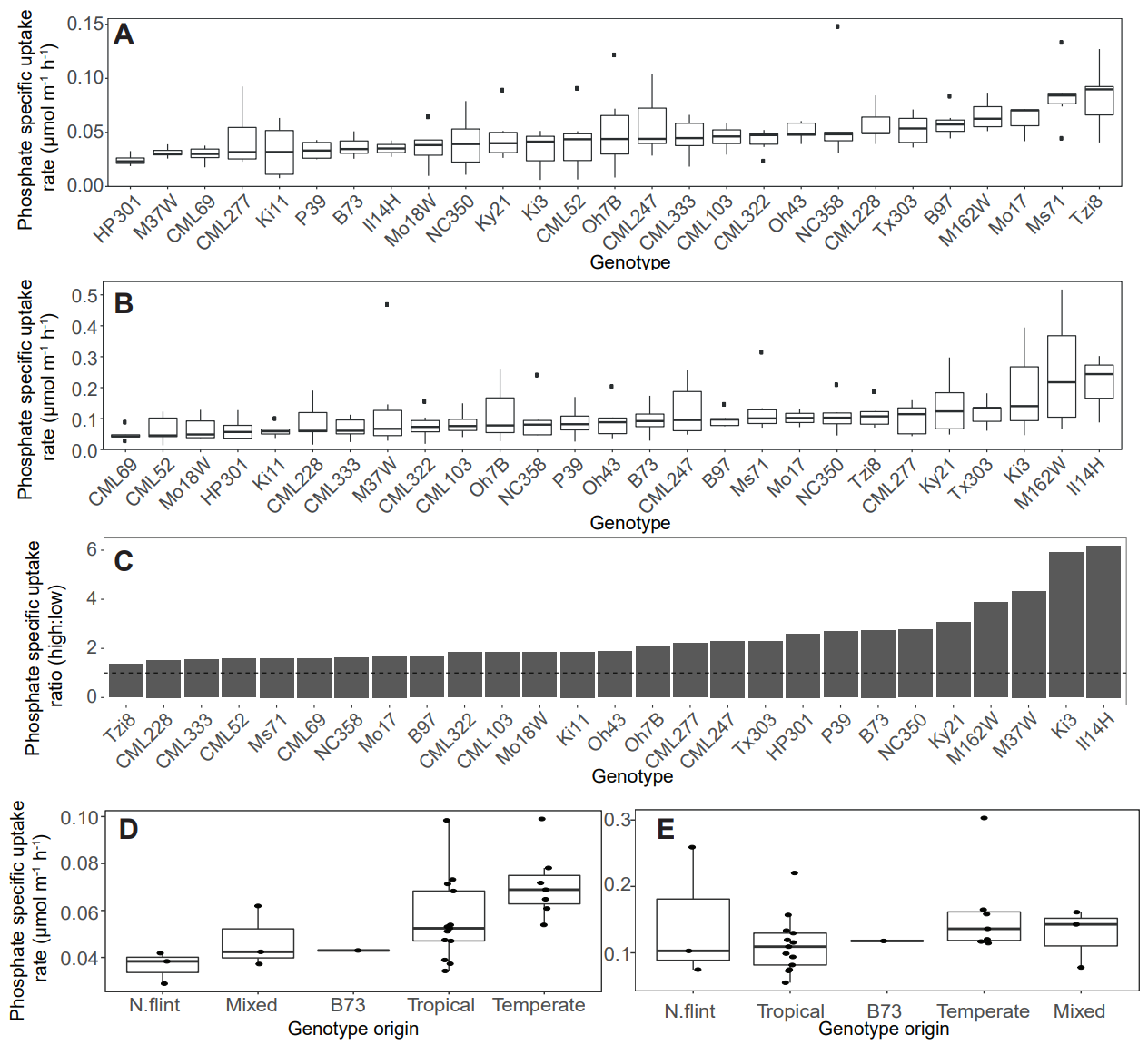


**Supplemental Figure S2.** Genetic diversity among nested association mapping (NAM) population founder lines and broad group classification for specific potassium uptake rates. Specific potassium uptake rates from solution concentrations of (A) 100 μM and (B) 1 mM. (C) The net specific potassium uptake rate ratio between the solutions, with a ratio >1 (dotted line) representing a greater uptake rate in the high concentration compared to the low concentration. The specific potassium uptake rates by broad group classification in the solution concentrations of (D) 25 μM and (E) 250 μM. Boxplots show the median values (center horizontal lines), upper and lower quartiles (box length), minimum and maximum values (whiskers), mean values, (dots within whiskers), and, outliers (dots outside of the whiskers).


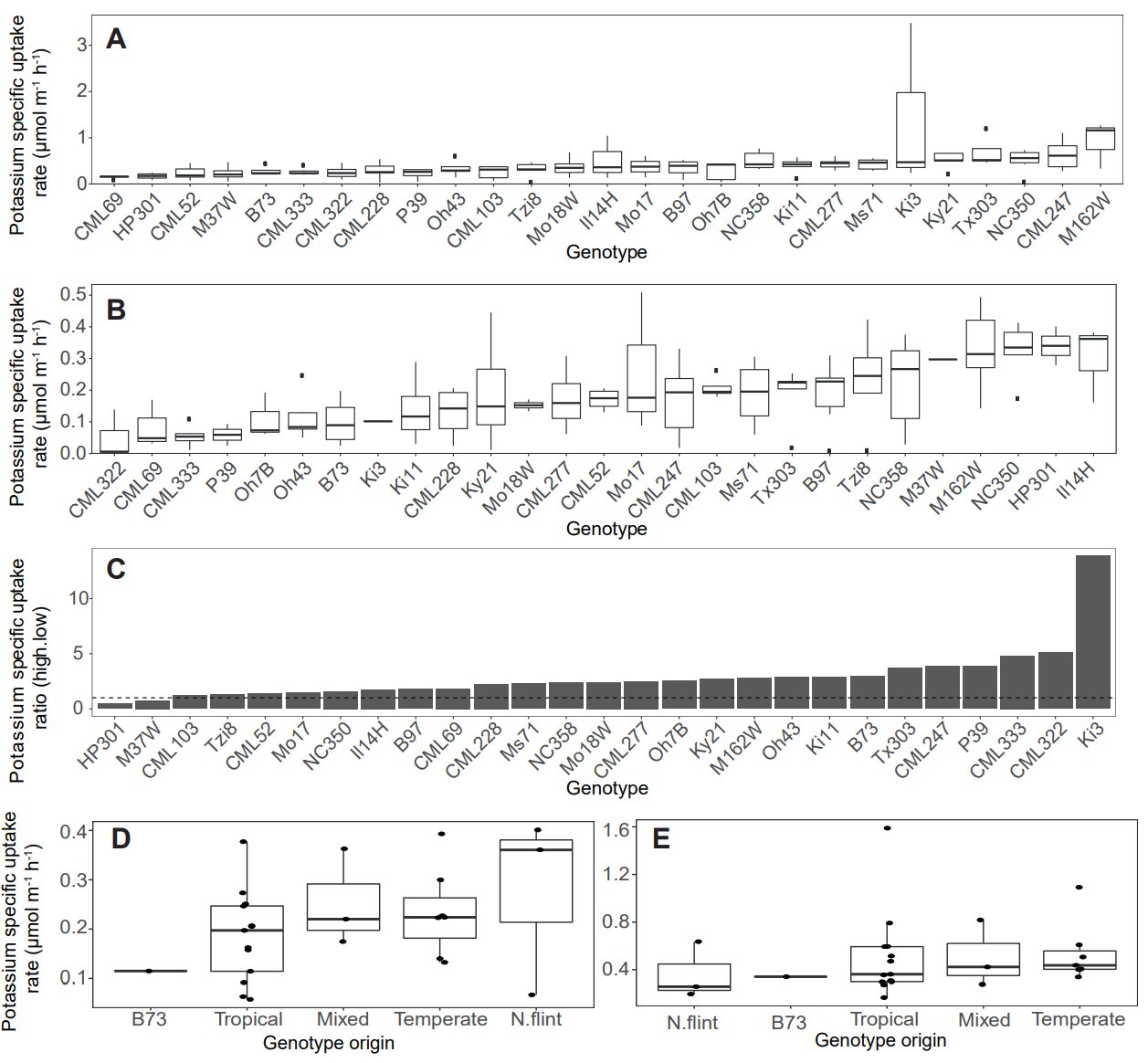


**Supplemental Figure S3.** Genetic diversity among nested association mapping (NAM) population founder lines and broad group classification for specific sulfate uptake rates. Specific sulfate uptake rates from solution concentrations of (A) 25 μM and (B) 250 μM with a significant genotype × concentration interaction in ANOVA (P < 0.05) indicated by the change in genotype ranking. (C) The net specific sulfate uptake rate ratio between the solutions, with a ratio >1 (dotted line) representing a greater uptake rate in the high concentration compared to the low concentration. The specific sulfate uptake rates by broad group classification in the solution concentrations of (D) 25 μM and (E) 250 μM. Boxplots show the median values (center horizontal lines), upper and lower quartiles (box length), minimum and maximum values (whiskers), mean values, (dots within whiskers), and, outliers (dots outside of the whiskers).


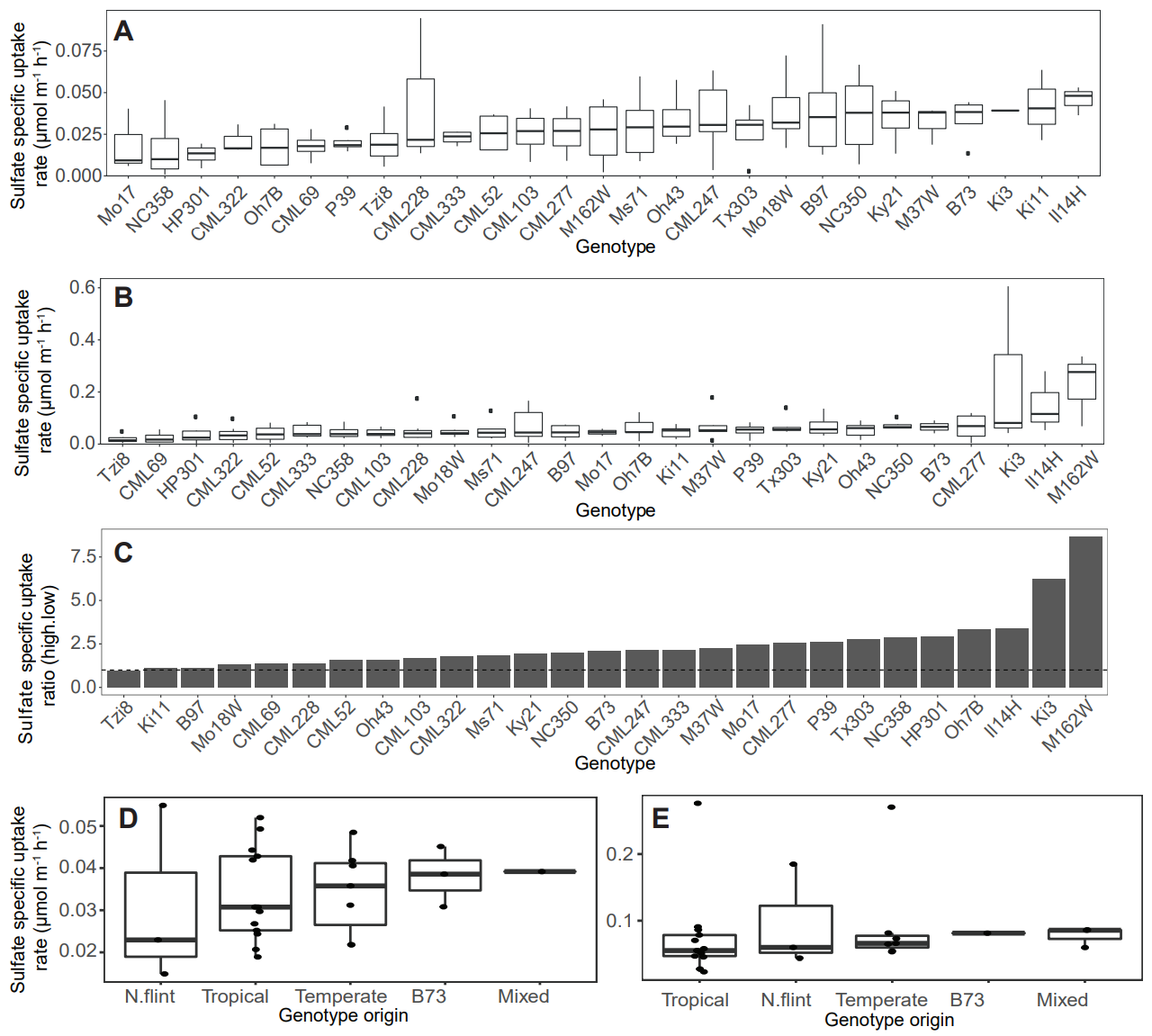


**Supplemental Figure S4.** Genetic diversity among nested association mapping (NAM) population founder lines and broad group classification for specific ammonium uptake rates. Specific ammonium uptake rates from solution concentrations of (A) 100 μM and (B) 1 mM. (C) The net specific ammonium uptake rate ratio between the solutions, with a ratio >1 (dotted line) representing a greater uptake rate in the high concentration compared to the low concentration. The specific ammonium uptake rates by broad group classification in the solution concentrations of (D) 25 μM and (E) 250 μM. Boxplots show the median values (center horizontal lines), upper and lower quartiles (box length), minimum and maximum values (whiskers), mean values, (dots within whiskers), and, outliers (dots outside of the whiskers).


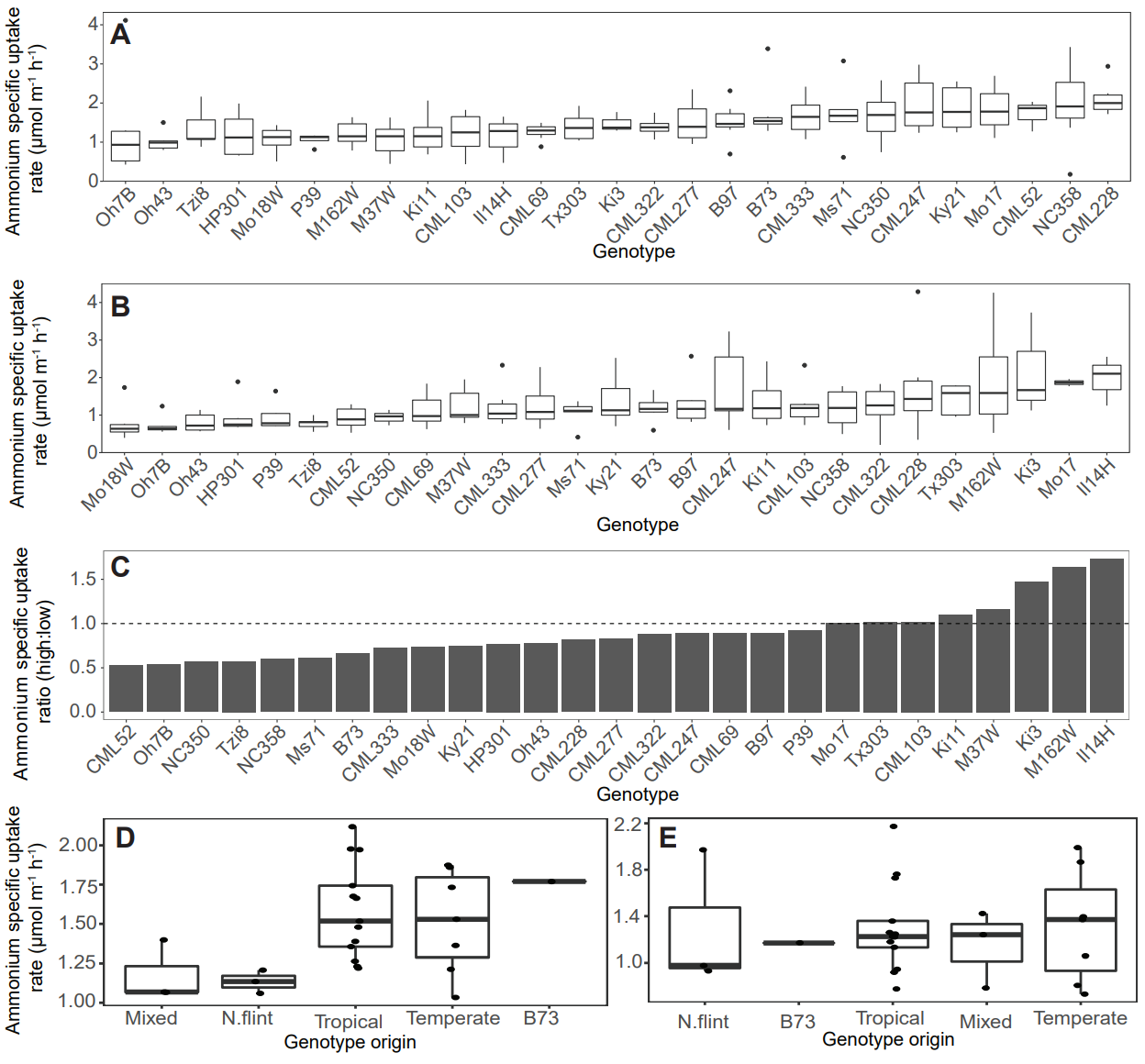


**Supplemental Figure S5**. Broad-sense heritability scores for specific nutrient uptake rates, respiration rate and length parameters amongst the nested association mapping (NAM) population founder lines. Heritability scores were calculated using the mean number of experimental replications. Broad-sense heritability scores >0.3 are commonly regarded as heritable.


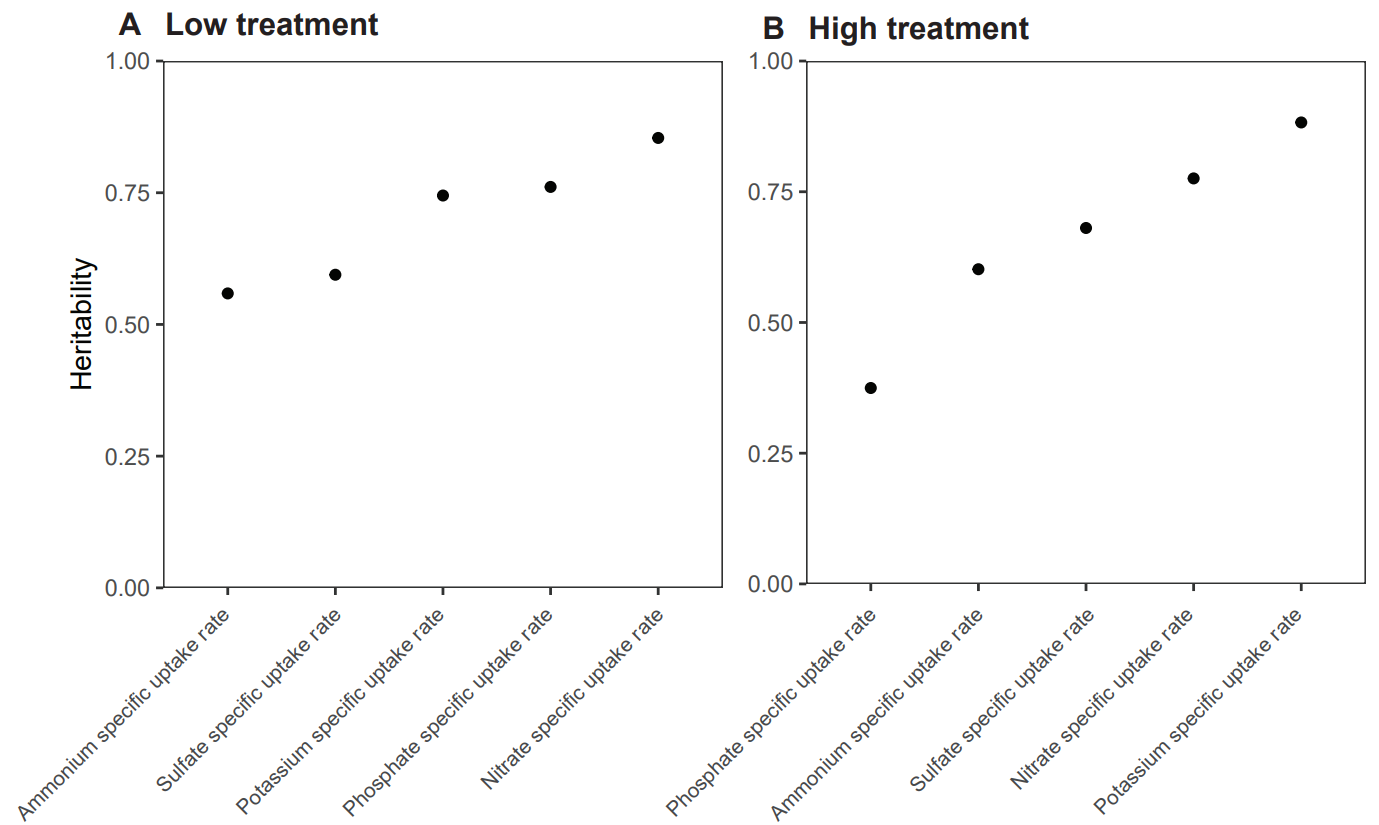


**Supplemental Figure S6.** Linear regression analyses between the specific nutrient uptake rates of the low and high treatments among the nested association mapping (NAM) population founder lines. (A) Nitrate. (B) Phosphate. (C) Potassium. (D) Sulfate. (E) Ammonium. Each dot represents the mean of each genotype. Signiﬁcant relationships are depicted with a full red line and non-signiﬁcant relationships with a dashed red line. The grey bar represents a 95% confidence region.


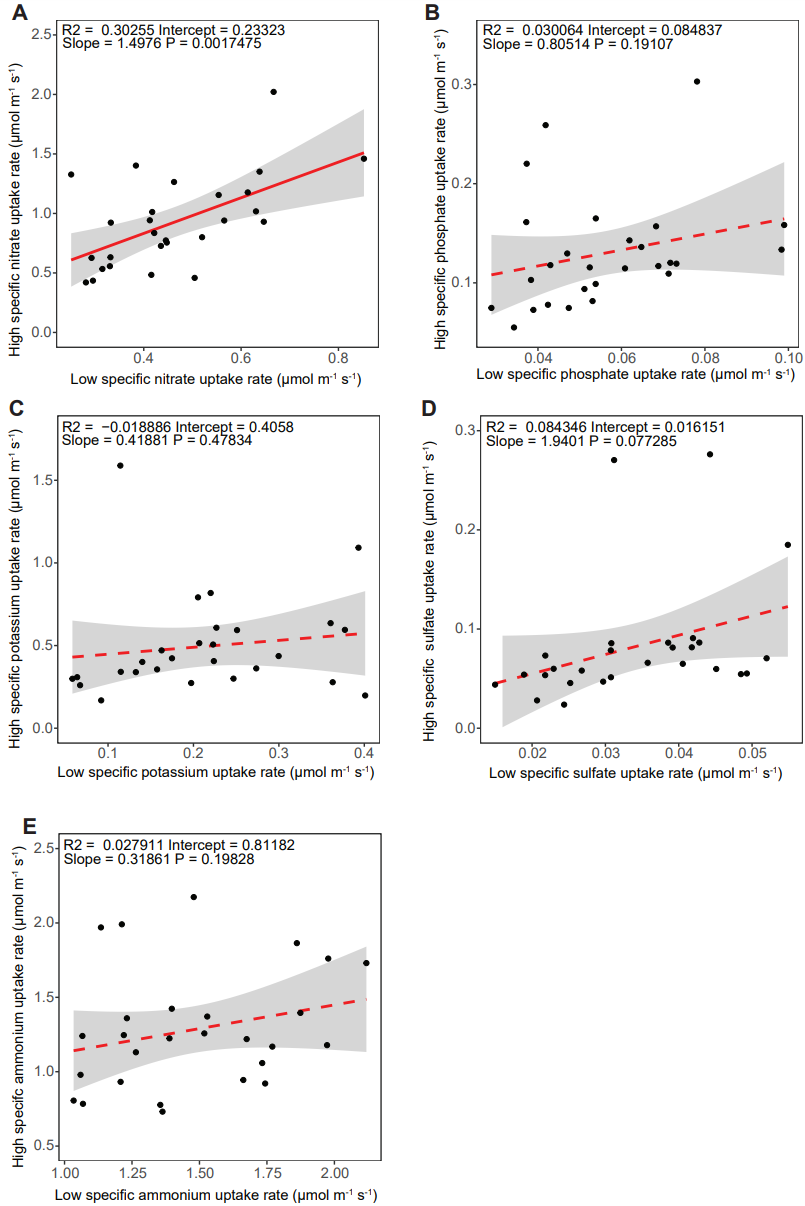


**Supplemental Figure S7**. Correlation and interaction of plant traits among the nested association mapping (NAM) population founder lines under the low concentration solution. (A) PCA ordination of extracted plant traits under the low nutrient treatments of the NAM population founders. Arrows indicate directions of loadings for each trait and are color coded by contribution to the percent variation of the component. (B) Correlation matrix for specific root nutrient uptake, respiration and length parameters under the high nutrient treatment. Correlations are color coded from strong positive correlation in red to strong negative correlation in blue with no correlation shown in white.


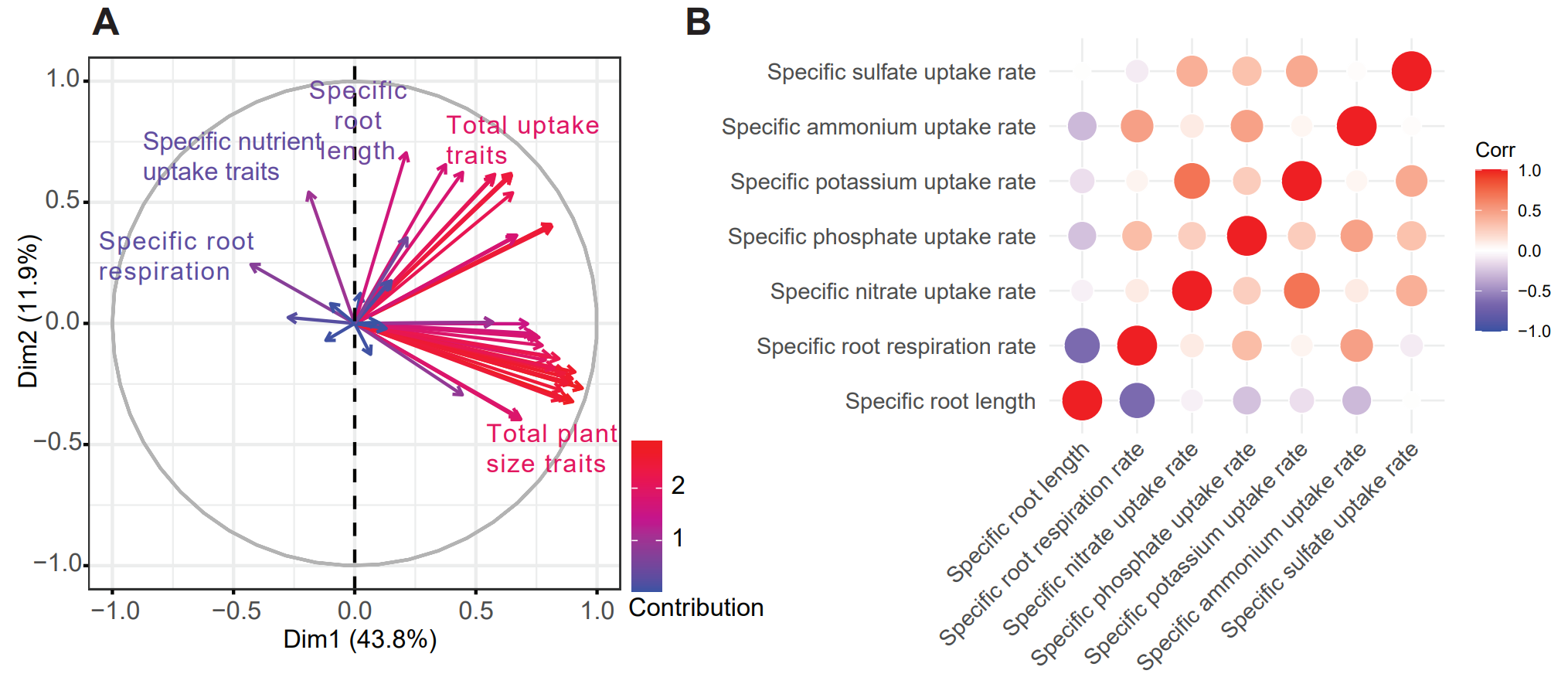


**Supplemental Figure S8**. The net specific nutrient uptake rate ratio between potassium and phosphate among the nested association mapping (NAM) population founder lines. (A) High concentration level. (B) Low concentration level (P < 0.01). A ratio of above 1 represents a higher proportion of potassium uptake compared to phosphate. Boxplots show the median values (center horizontal lines), upper and lower quartiles (box length), minimum and maximum values (whiskers), mean values, (dots within whiskers), and, outliers (dots outside of the whiskers). ANOVA results for all nutrient combinations are in Table S2.


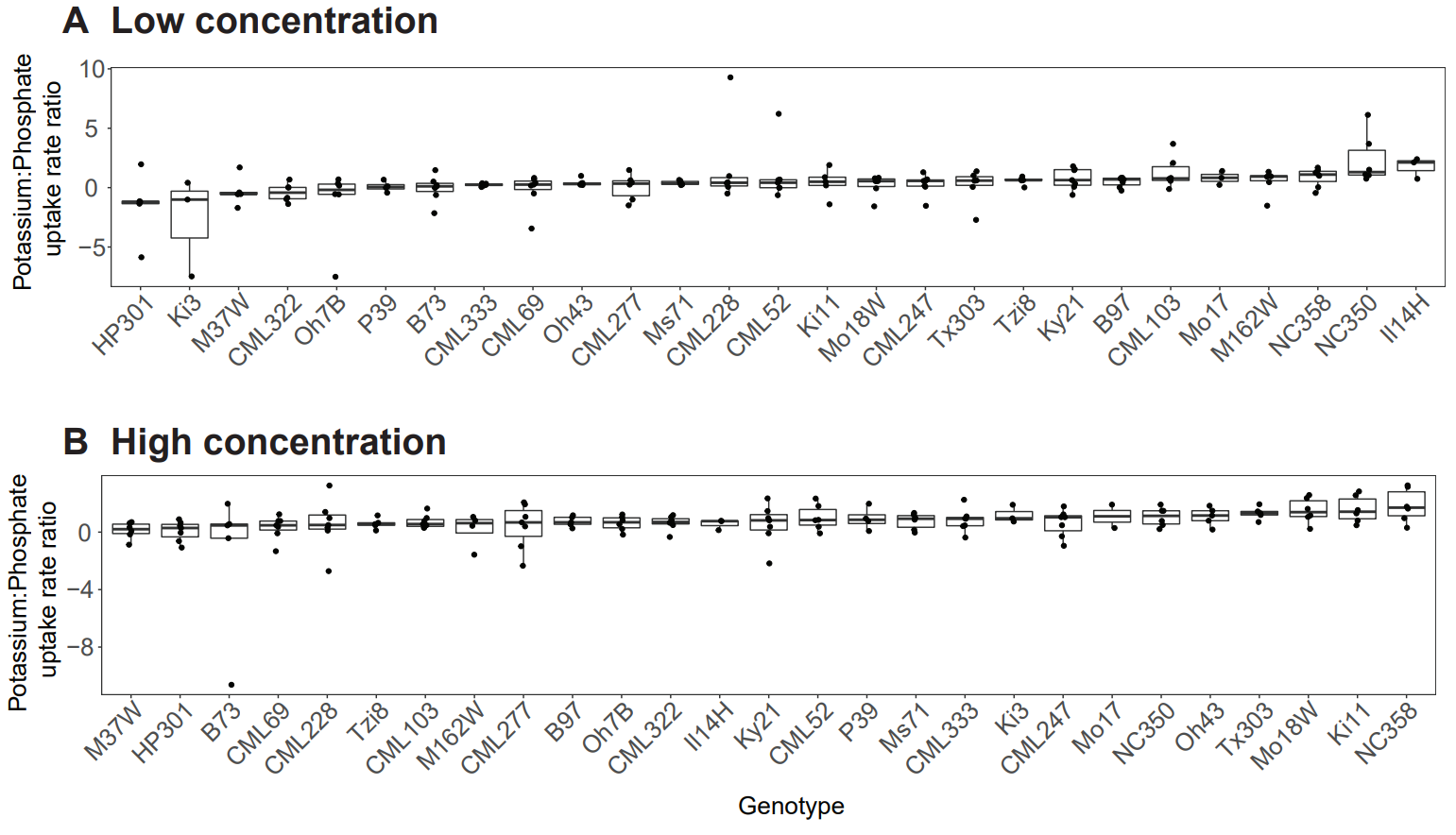


**Supplemental Figure S9.** Linear regression analyses between specific nutrient uptake rate and specific root respiration rate in the low and high treatments among the nested association mapping (NAM) population founder lines. (AB) Phosphate. (CD) Potassium. (EF) Sulfate. (GH) Ammonium. Signiﬁcant relationships are depicted with a full line and non-signiﬁcant relationships with a dashed line.


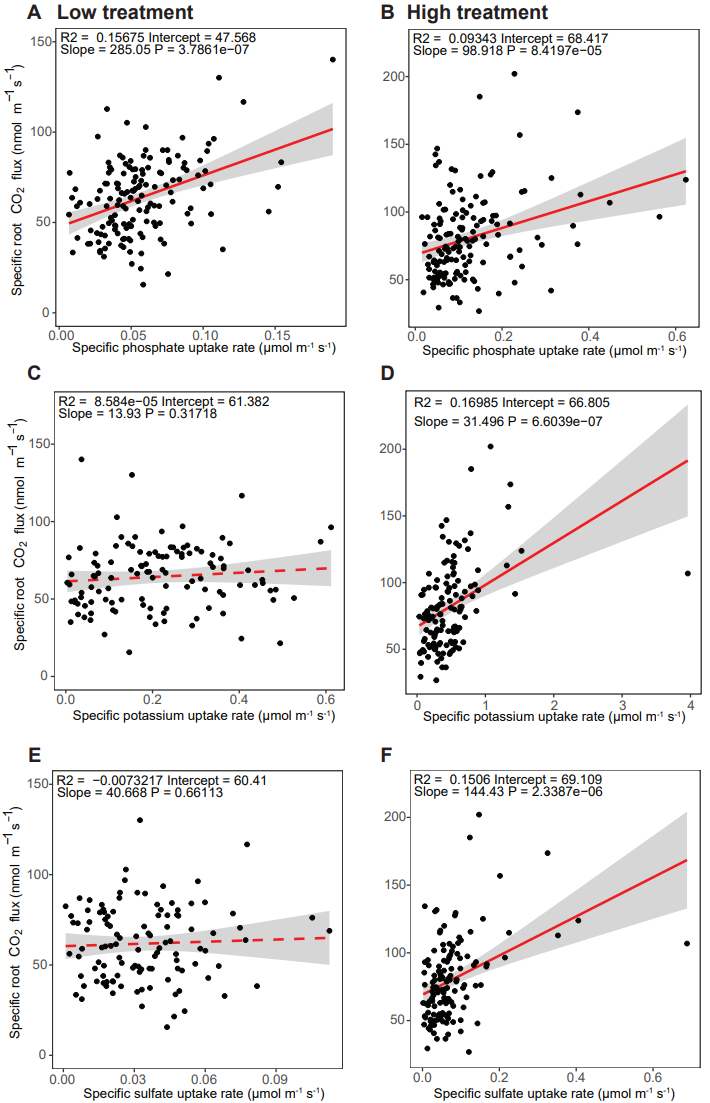


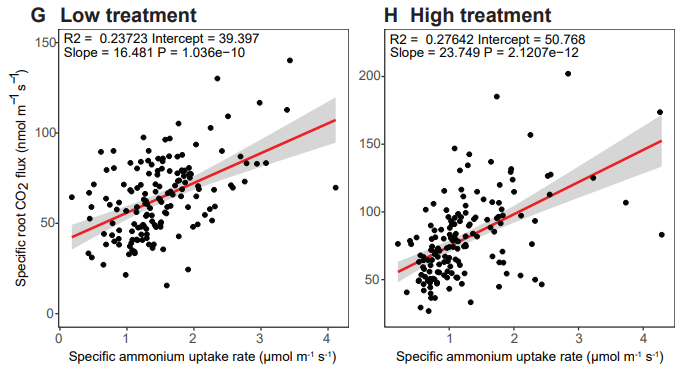


**Supplemental Table S1**. Definition of plant traits measured.

| Trait category | Trait description | Method | Units |
| --- | --- | --- | --- |
| Total uptake traits | Total ammonium uptake | Ion chromatography | umol plant^-1^ |
|  | Total nitrate uptake | Ion chromatography | umol plant^-1^ |
|  | Total phosphate uptake | Ion chromatography | umol plant^-1^ |
|  | Total potassium uptake | Ion chromatography | umol plant^-1^ |
|  | Total sulfate uptake | Ion chromatography | umol plant^-1^ |
| Specific uptake traits | Specific ammonium uptake rate (length) | - | umol m^-1^ h^-1^ |
|  | Specific nitrate uptake rate (length) | - | umol m^-1^ h^-1^ |
|  | Specific phosphate uptake rate (length) | - | umol m^-1^ h^-1^ |
|  | Specific potassium uptake rate (length) | - | umol m^-1^ h^-1^ |
|  | Specific sulfate uptake rate (length) | - | umol m^-1^ h^-1^ |
|  | Specific ammonium uptake rate (mass) | - | umol g^-1^ h^-1^ |
|  | Specific nitrate uptake rate (mass) | - | umol g^-1^ h^-1^ |
|  | Specific phosphate uptake rate (mass) | - | umol g^-1^ h^-1^ |
|  | Specific potassium uptake rate (mass) | - | umol g^-1^ h^-1^ |
|  | Specific sulfate uptake rate (mass) | - | umol g^-1^ h^-1^ |
| Specific uptake ratio | Ammonium:Phosphate uptake rate ratio (length) | - | - |
|  | Ammonium:Potassium uptake rate ratio (length) | - | - |
|  | Ammonium:Sulfate uptake rate ratio (length) | - | - |
|  | Nitate:Ammonium uptake rate ratio (length) | - | - |
|  | Nitate:Phosphate uptake rate ratio (length) | - | - |
|  | Nitate:Potassium uptake rate ratio (length) | - | - |
|  | Nitate:Sulfate uptake rate ratio (length) | - | - |
|  | Phosphate:Sulfate uptake rate ratio (length) | - | - |
|  | Potassium:Phosphate uptake rate ratio (length) | - | - |
|  | Potassium:Sulfate uptake rate ratio (length) | - | - |
|  | Ammonium:Phosphate uptake rate ratio (mass) | - | - |
|  | Ammonium:Potassium uptake rate ratio (mass) | - | - |
|  | Ammonium:Sulfate uptake rate ratio (mass) | - | - |
|  | Nitate:Ammonium uptake rate ratio (mass) | - | - |
|  | Nitate:Phosphate uptake rate ratio (mass) | - | - |
|  | Nitate:Potassium uptake rate ratio (mass) | - | - |
|  | Nitate:Sulfate uptake rate ratio (mass) | - | - |
|  | Phosphate:Sulfate uptake rate ratio (mass) | - | - |
|  | Potassium:Phosphate uptake rate ratio (mass) | - | - |
|  | Potassium:Sulfate uptake rate ratio (mass) | - | - |
| Total root size traits | Total root mass | - | g plant^-1^ |
|  | Total root CO_2_ flux | LI-8100A | nmol plant^-1^ s^-1^ |
|  | Total length of all roots | WinRhizo | cm plant^-1^ |
|  | Total length of seminal roots | WinRhizo | cm plant^-1^ |
|  | Total length of lateral roots | WinRhizo | cm plant^-1^ |
| Specific root size traits | Specific root CO_2_ flux (length) | LI-8100A | nmol m^-1^ s^-1^ |
|  | Specific root CO_2_ flux (mass) | LI-8100A | nmol g^-1^ s^-1^ |
|  | Specific root length | LI-8100A | m g^-1^ |
| Total shoot size traits | Leaf dry mass | - | g plant^-1^ |
|  | Leaf length | FIJI macro | cm plant^-1^ |
|  | Leaf surface area | FIJI macro | cm^2^ plant^-1^ |
|  | Total leaf N | Dumas method | g plant^-1^ |
|  | Total leaf protein | Dumas method | g plant^-1^ |
| Specific shoot size traits | Leaf N percent | Dumas method | % |
|  | Leaf protein percent | Dumas method | % |

**Supplemental Table S2.** Analysis of variance (ANOVA) table for the ion uptake and plant phenotypic traits as influenced by the high and low nutrient regimes.

|  |  | Source of variation | | |
| --- | --- | --- | --- | --- |
| Trait category | Traits | Genotype | Treatment | Geno*Treatment |
| Total uptake | Ammonium total uptake (µmol plant^-1^) | 1.93 ** | 285.23 *** | 1.21 ns |
|  | Nitrate total uptake (µmol plant^-1^) | 3.43 *** | 389.31 *** | 2.53 *** |
|  | Phosphate total uptake (µmol plant^-1^) | 1.40 ns | 315.09 *** | 1.08 ns |
|  | Potassium total uptake (µmol plant^-1^) | 2.42 *** | 152.48 *** | 1.93 ** |
|  | Sulfate total uptake (µmol plant^-1^) | 1.58 * | 205.70 *** | 1.18 ns |
| Specific uptake | Specific ammonium uptake rate (µmol g^-1^ h^-1^) | 3.48 *** | 10.58 ** | 1.72 * |
|  | Specific ammonium uptake rate (µmol m^-1^ h^-1^) | 2.22 ** | 6.97 ** | 1.19 ns |
|  | Specific nitrate uptake rate (µmol g^-1^ h^-1^) | 4.22 *** | 75.40 *** | 1.78 * |
|  | Specific nitrate uptake rate (µmol m^-1^ h^-1^) | 3.49 *** | 98.58 *** | 1.55 * |
|  | Specific phosphate uptake rate (µmol g^-1^ h^-1^) | 3.16 *** | 77.21 *** | 1.95 ** |
|  | Specific phosphate uptake rate (µmol m^-1^ h^-1^) | 2.39 *** | 88.87 *** | 1.51 ns |
|  | Specific potassium uptake rate (µmol g^-1^ h^-1^) | 3.78 *** | 59.46 *** | 1.98 ** |
|  | Specific potassium uptake rate (µmol m^-1^ h^-1^) | 2.29 ** | 51.60 *** | 1.56 ns |
|  | Specific sulfate uptake rate (µmol g^-1^ h^-1^) | 3.18 *** | 48.11 *** | 2.49 *** |
|  | Specific sulfate uptake rate (µmol m^-1^ h^-1^) | 1.95 ** | 47.65 *** | 1.83 * |
| Specific uptake ratio | Ammonium:Phosphate ratio | 1.50 ns | 1.21 ns | 31.75 *** |
|  | Ammonium:Potassium ratio | 1.05 ns | 1.05 ns | 0.68 ns |
|  | Ammonium:Sulfate ratio | 0.74 ns | 0.69 ns | 0.06 ns |
|  | Nitate:Ammonium ratio | 2.17 ** | 1.29 ns | 119.62 *** |
|  | Nitate:Phosphate ratio | 1.20 ns | 1.35 ns | 0.47 ns |
|  | Nitate:Potassium ratio | 1.20 ns | 1.35 ns | 0.47 ns |
|  | Nitate:Sulfate ratio | 0.70 ns | 0.57 ns | 0.03 ns |
|  | Phosphate:Sulfate ratio | 0.79 ns | 0.68 ns | 0.04 ns |
|  | Potassium:Phosphate ratio | 2.11 ** | 1.44 ns | 5.65 * |
|  | Potassium:Sulfate ratio | 0.85 ns | 0.55 ns | 2.36 ns |
| Total shoot size | Leaf area (cm^2^ plant^-1^) | 5.70 *** | 0.64 ns | 0.82 ns |
|  | Leaf length (cm plant^-1^) | 4.48 *** | 0.47 ns | 1.04 ns |
|  | Leaf weight (g plant^-1^) | 4.55 *** | 0.02 ns | 0.81 ns |
|  | Stem weight (g plant^-1^) | 5.49 *** | 0.06 ns | 0.74 ns |
|  | Shoot weight (g plant^-1^) | 5.11 *** | 0.04 ns | 0.76 ns |
| Total root size | Root weight (g plant^-1^) | 6.91 *** | 0.02 ns | 0.66 ns |
|  | Root CO_2_ flux (nmol plant^-1^ s^-1^) | 6.32 *** | 22.01 *** | 1.18 ns |
|  | Root count lateral | 8.45 *** | 0.32 ns | 0.69 ns |
|  | Root count secondary lateral | 3.63 *** | 0.00 ns | 0.73 ns |
|  | Root count seminal | 5.74 *** | 3.72 ns | 1.03 ns |
|  | Root count total | 3.67 *** | 0.01 ns | 0.73 ns |
|  | Root length lateral (cm plant^-1^) | 4.22 *** | 0.48 ns | 0.69 ns |
|  | Root length secondary lateral (cm plant^-1^) | 5.18 *** | 0.08 ns | 0.78 ns |
|  | Root length seminal (cm plant^-1^) | 7.39 *** | 0.23 ns | 0.64 ns |
|  | Root length total (cm plant^-1^) | 3.37 *** | 0.28 ns | 0.71 ns |
|  | Root projected area lateral (cm^2^ plant^-1^) | 5.14 *** | 0.48 ns | 0.67 ns |
|  | Root projected area secondary lateral (cm^2^ plant^-1^) | 4.93 *** | 0.04 ns | 0.81 ns |
|  | Root projected area seminal (cm^2^ plant^-1^) | 7.63 *** | 0.16 ns | 0.68 ns |
|  | Root projected area total (cm^2^ plant^-1^) | 4.42 *** | 0.23 ns | 0.68 ns |
|  | Root projected volume lateral (cm^3^ plant^-1^) | 6.50 *** | 0.33 ns | 0.69 ns |
|  | Root projected volume secondary lateral (cm^3^ plant^-1^) | 4.74 *** | 0.02 ns | 0.84 ns |
|  | Root projected volume seminal (cm^3^ plant^-1^) | 7.66 *** | 0.08 ns | 0.74 ns |
|  | Root projected volume total (cm^3^ plant^-1^) | 6.01 *** | 0.06 ns | 0.71 ns |
|  | Root surface area lateral (cm^2^ plant^-1^) | 5.14 *** | 0.48 ns | 0.67 ns |
|  | Root surface area secondary lateral (cm^2^ plant^-1^) | 4.93 *** | 0.04 ns | 0.81 ns |
|  | Root surface area seminal (cm^2^ plant^-1^) | 7.63 *** | 0.16 ns | 0.68 ns |
|  | Root surface area total (cm^2^ plant^-1^) | 4.42 *** | 0.23 ns | 0.68 ns |
| Specific root traits | Specific root CO_2_ flux (nmol g^-1^ s^-1^) | 3.11 *** | 57.75 *** | 1.24 ns |
|  | Specific root CO_2_ flux (nmol m^-1^ s^-1^) | 4.86 *** | 44.78 *** | 0.87 ns |
|  | Specific root length (m g^-1^) | 21.41 *** | 0.17 ns | 0.56 ns |
|  | ***denotes p<0.001; ** p<0.01; *p<0.05; ns = not significant | | | |

**Supplemental Table S3.** Primer sequences for qPCR analysis

| GeneID | Putative gene | Forward | Reverse | Reference |
| --- | --- | --- | --- | --- |
| Zm00001d034782 | Ammonium Transporter | AAAGACGGCATACCGAAGAATC | AAACTGCACAAGGACGAGGAA |  |
| Zm00001d018421 | C2C2-GATA Transcription Factor | AGCATCCATCCAATCCCAATC | TGCAGCAACCACTCTGACTCA |  |
| Zm00001d029963 | MYB-related Transcription Factor | GGGTTGATGGGTCCCTGTCT | CCTCGGATTGGAACACGAA |  |
| Zm00001d046679 | Potassium Transporter | CATGTGCATCAAGAACGTACCA | CTCCCAACGAGGAACCTCTCT |  |
| Zm00001d020938 | Protein Coding Transcript | GAGAGAGAGCTAGTGAGGACGAGAA | CGACAATCGTCGCCAAGATT |  |
| Zm00001d006293 | Protein Coding Transcript 2 | GTCCCTTAAGATCACAGCATTCCT | TTCATGTGCATTGAACAGAGCAT |  |
| Zm00001d021442 | Protein Coding Transcript 3 | TGTGCCACCAAGTCCCAGTT | CCTAACCTGAGCAACAGATCGA |  |
| Zm00001d046838 | Putative receptor-like protein kinase family protein | CGACGGTCTCGGATGTGAA | CTGGACCTGTGACGGGAGTT |  |
| Zm00001d020826 | BHLH Transcription Factor | ACTGAGGAAAAGCTCGTGGC | ACCGCCGAAACAGACAGAAT |  |
| Zm00001d051879 | HK1-NAK | CATTCGAAGGGATCTCTGAGGTT | CCAGATGAGGATGCACCGTACT | y |
| Zm00001d002944 | RPN-primer | CGTTGCCTTCATTGCGTATCT | CCAACAGGCCTATGATGATTTTC | y |
| Zm00001d044172 | SGT1-primer | CGTCCACCCATAGTGCCATGAG | ACGGCAAGTAGCACTCAGACAC | y |
