# Supplementary figures and images for: A multiple ion-uptake phenotyping platform reveals shared mechanisms affecting nutrient uptake by roots
Source: Plant Physiol. 2020 Dec 29;185(3):781–95. doi: 10.1093/plphys/kiaa080 (PMC8133564; doi:10.1093/plphys/kiaa080)

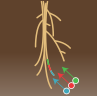

Supplement: kiaa080_Supplementary_Data [file kiaa080_supplementary_data.zip › pp.01265.2020-s05.jpg]
