# Supplementary material for: A multiple ion-uptake phenotyping platform reveals shared mechanisms affecting nutrient uptake by roots
Source: Plant Physiol. 2020 Dec 29;185(3):781–95. doi: 10.1093/plphys/kiaa080 (PMC8133564; doi:10.1093/plphys/kiaa080)
Supplement: kiaa080_Supplementary_Data [file kiaa080_supplementary_data.zip › pp.01265.2020-s01.docx]

Dear Editor,

Thank you for reviewing our manuscript and providing valuable feedback which will no doubt improve the final version for Plant Physiology’s readership. It is encouraging to see the positive reception of the novelty and value of the platform from both reviewers. This platform took us two years to optimize through many time-consuming preliminary studies, so the work presented was not a pilot study. Again, we would like to re-emphasize the novelty of measuring such a wide diversity of traits which opens up many new avenues of investigation, with this study representing one of the most ‘phenotypically dense’ datasets in existence.

As suggested, we have resubmitted as a Breakthrough Technologies article by including a little more emphasis on the platform, such as by naming it in the abstract, added hardware schematics in Fig 1, and in the results by first describing the platform. Still, we think this platform allowed us to make valuable insights into potentially exciting biological discoveries, which we understand that we did not extensively validate here. We feel we wrote all speculative discussion with carefully chosen words (like use of ‘may’) and highlighting specific contexts (like only to the specific age plants we studied) that limited over-extrapolation. We tried to re-emphasize which points are more or less speculative by responding to their specific comments and re-writing as necessary. Still, we feel that providing these biological insights, made possible by this platform, is an important contribution to the literature of an under-studied area and so hope we can be allowed some careful speculation that might spur future research beyond our own capabilities, which do not include many molecular techniques since we focus on whole plant physiology. Definitely, we believe that speculation is warranted on how demonstrating heritable genetic variation for uptake rates and how they relate *could* have relevance for breeding and agriculture.

As far as details about the platform, we feel it was already extensively described in the methods, and we already provided DOIs (links) to the schematics and parts list on Zenodo, as well as the protocols. Actually then, we believe that we’ve gone through more lengths than typical to ensure the system is reproducible by other groups. As noted above, we added some text to the results to highlight this to the reader, which may have been lost because the requested manuscript format places methods at the end, even for methods-focused articles.

We have provided a point by point response to each of the issues that the reviewers raised in the separate reply to reviewers.

Kind regards,

Larry York, Marcus Griffiths, and colleagues
